# Quantification of *N*-acetylcysteamine activated methylmalonate incorporation into polyketide biosynthesis

**DOI:** 10.3762/bjoc.9.75

**Published:** 2013-04-05

**Authors:** Stephan Klopries, Uschi Sundermann, Frank Schulz

**Affiliations:** 1 Fakultät für Chemie, Technische Universität Dortmund, Otto-Hahn-Str. 6, 44221 Dortmund, Germany; 2 Abteilung für Chemische Biologie, Max-Planck-Institut für Molekulare Physiologie, Otto-Hahn-Str. 11, 44227 Dortmund, Germany

**Keywords:** biosynthesis, coenzyme A, malonic acid, polyketide, polyketide synthase

## Abstract

Polyketides are biosynthesized through consecutive decarboxylative Claisen condensations between a carboxylic acid and differently substituted malonic acid thioesters, both tethered to the giant polyketide synthase enzymes. Individual malonic acid derivatives are typically required to be activated as coenzyme A-thioesters prior to their enzyme-catalyzed transfer onto the polyketide synthase. Control over the selection of malonic acid building blocks promises great potential for the experimental alteration of polyketide structure and bioactivity. One requirement for this endeavor is the supplementation of the bacterial polyketide fermentation system with tailored synthetic thioester-activated malonates. The membrane permeable *N*-acetylcysteamine has been proposed as a coenzyme A-mimic for this purpose. Here, the incorporation efficiency into different polyketides of *N*-acetylcysteamine activated methylmalonate is studied and quantified, showing a surprisingly high and transferable activity of these polyketide synthase substrate analogues in vivo.

## Introduction

**Figure 1:**
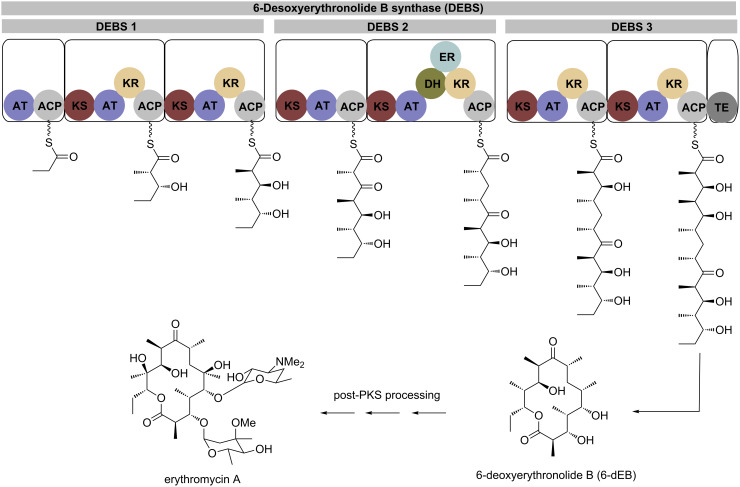
The most intensively studied PKS, deoxyerythronolide B synthase (DEBS), which catalyzes the key steps in the biosynthesis of the antibiotic erythromycin. DEBS catalyzes the extension of a propionate starter unit with six equivalents of methylmalonyl-coenzyme A (MM-CoA). After six rounds of decarboxylative Claisen condensations and varying degrees of reduction of the initially formed β-keto thioesters, the polyketide core of erythromycin is released from the enzyme via a terminal esterase [6–8]. Abbreviations: AT: acyltransferase, ACP: acyl carrier protein, KS: ketosynthase, KR: ketoreductase, DH: dehydratase, ER: enoylreductase, TE: thioesterase.

Polyketides are ubiquitous natural products and find widespread application in current medicine and agriculture. Polyketide synthases (PKS), giant multienzyme complexes, play a pivotal role in their biosynthesis. PKS generate molecular complexity and diversity through a number of stepwise condensations in analogy to fatty acid synthases but with optional and varying degrees of reduction in each step (Figure 1) [1–3]. Additional diversity is introduced by the incorporation of different carboxylic acid starter units and a range of different extender units, usually coenzyme A-activated malonic acid derivatives, with varying substituents at C-2 [4–5].

Current experiments to generate biosynthetic polyketide diversity focus on different aspects of the biosynthetic reaction cascade. Mainly by genetic replacement or deletion of various fragments of PKS, alterations in chain length [9–11], redox pattern [12–14], stereochemistry [15–17], and starter unit diversity [18–20] were in several instances introduced, giving rise to a significant number of polyketide derivatives to date. However, the accessible extender unit diversity is currently restricted to a small number of different malonate units and changes in the polyketide side chain pattern are highly sought after [4,17,21–26]. Different strategies can be pursued to introduce non-native extender units into the PKS machinery. They rely on the replacement of an acyltransferase domain of a given PKS module with another domain possessing different substrate specificity by using various different strategies. Subsequently, thioester activated non-native malonate derivatives can be synthesized by additional heterologously expressed biosynthetic pathways leading to the respective derivative of malonyl-CoA [17] or malonyl-ACP [27], which are supplied in vivo to the mutated PKS. Another option is the exogenous supply of the malonate derivative [28], typically activated as *N*-acetylcysteamine thioesters (malonyl-SNAC) [29–40]. Despite its apparent simplicity, the latter option is not well characterized for in vivo applications. Especially the bioavailability and the coupled acceptance efficiency to the corresponding CoA-esters remain to be clarified. This has recently become highly relevant, as the first example for an acyltransferase domain with artificially broadened substrate specificity has been constructed and introduced in the biosynthetic pathway towards erythromycin in place of the native AT6 domain [39]. This has led to the formation of 2-propargylerythromycin through the incorporation of 2-propargylmalonate into the biosynthetic pathway, activated as SNAC-thioester and supplied to the bacterial fermentation. In this step, the exogenously supplied synthetic building block competes with the endogenous methylmalonyl-CoA (MM-CoA) for acceptance by the same enzyme domain; hence, the result of this experiment was the formation of the wild-type erythromycin product as a mixture with the new propargylated derivative. It is now of interest to judge the efficiency of SNAC- versus CoA-activation. This can show to which extent the activation influences the choice of the extender units in the case that a promiscuous acyltransferase domain can catalyze the incorporation of a natural and a non-natural building block.

We here report the characterization of deuterated methylmalonyl-SNAC ester incorporation into polyketides in vivo and therefore in direct competition to endogenous MM-CoA, to determine the relative incorporation efficiency and the concentrations required to saturate polyketide synthases with the artificial extender unit donor. This determines the impact of SNAC- versus CoA-activation and gives rise to rapid optimization of feeding experiments using exogenously supplied malonate-derivatives. These experiments point the way towards the incorporation of non-native malonate derivatives into biosynthetic pathways en route to new polyketide derivatives.

## Results and Discussion

### Synthesis of D_3_-labeled malonyl-SNAC-esters

The incorporation of artificially activated exogenous methylmalonate is most straightforwardly monitored by LC/ESI–MS. This requires stable-isotope-labeled material, preferably a D_3_-label.

**Scheme 1:**
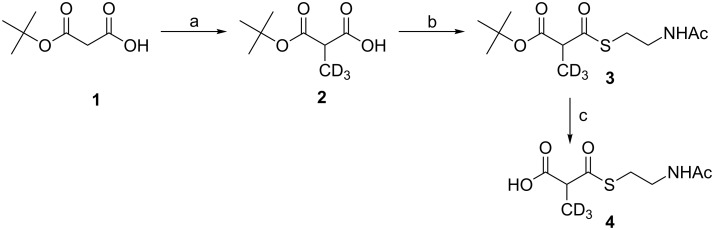
Synthesis of SNAC-activated D_3_-methylmalonate. a: 2.1 equiv [(CH_3_)_2_CH]_2_NLi, 1 equiv CD_3_I, abs. THF, −78 °C → rt, Ar, 18 h, 37%. b: 1.3 equiv *N*,*N*'-carbonyldiimidazole, 0.3 equiv DMAP, 1.5 equiv *N*-acetylcysteamine, abs. THF, 0 °C → rt, Ar, 18 h, 82%; c: 2.5 equiv TiCl_4_, CH_2_Cl_2_, Ar, 0 °C → rt, 6 h, then aq NaHCO_3_ (pH 8.0), quant. (by TLC and ^1^H NMR).

For adding exogenous malonates to bacterial fermentations, millimolar concentrations of the SNAC-activated substrates were expected to be required in the fermentation media, making reliable and preparative-scale synthetic procedures to obtain the SNAC-ester necessary. Hence, we decided to apply a modular approach to our synthesis with an intrinsic transferability to variously substituted malonates. After optimization, mono-*t*-Bu-protected malonate provided the best starting point for the synthesis (Scheme 1) [34].

The sterically demanding protective group prevented decarboxylation during the subsequent alkylation and thioesterification steps. Alkylation was achieved on a millimole-scale by using D_3_-iodomethane with LDA as base. After optimization, the isolated yield of **2** was 37%. However, when CH_3_I was used as electrophile for comparison, the yield reached 54% plus the additionally formed dialkylation product. The subsequent thioesterification with *N*-acetylcysteamine (SNAC) smoothly yielded compound **3** in 82% yield by using CDI to activate the malonic acid.

Enzymatic cleavage of the *tert*-butyl group was tested using the commercially available lipases CAL-A and CAL-B and gave only small amounts of the desired product at low reaction rates with no detectable decarboxylation of the products. In contrast, TFA-promoted cleavage resulted in quantitative decarboxylation even at low temperatures, low acid concentrations and short reaction times. Subsequently, Lewis-acid-mediated reactions were examined by using either ZnBr_2_ or TiCl_4_. Thus, 2.5 equivalents of TiCl_4_ rapidly lead to cleavage of the *tert*-butyl group, yielding compound **4** virtually byproduct free. The reaction product was found to be prone to decomposition upon concentration and storage. Work-up of the Lewis-acid-mediated *t*-Bu-cleavage reaction by the addition of aqueous NaHCO_3_ buffer (pH = 8.0), followed by removal of precipitated TiO_2_ and DCM led to an aqueous solution of the reaction product, which was analyzed by mass spectrometry and TLC. Separately, the reaction was quenched with a buffer of NaHCO_3_ in D_2_O to enable ^1^H NMR analysis of the reaction product. It was found that the TiCl_4_-based *t*-Bu-cleavage under these conditions gives quantitative conversion and no significant byproducts.

### Feeding experiments

**Figure 2:**
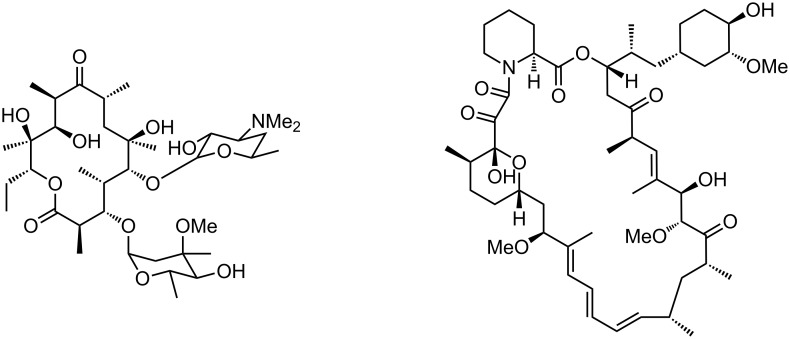
Structures of erythromycin (left) and rapamycin (right). In this experiment both compounds were labeled in their methyl side chains with deuterium.

The frequently studied biosynthetic routes of erythromycin [6–8] and rapamycin [41] were chosen as model pathways for the incorporation of the D_3_-label (Figure 2). These two polyketides are produced by bacteria from two different genera and possess different malonate incorporation patterns, thus providing significantly different test systems, albeit applying the same biosynthetic logic. By means of LC/ESI–MS analysis of the fermentation extracts, the incorporation was to be quantified in an erythromycin-producing strain of *Saccharopolyspora erythraea* (NRRL B-24071) and a rapamycin-producing strain of *Streptomyces hygroscopicus* (NRRL 5491) through the corresponding shift in the isotope ratio.

For feeding experiments, the carbonate buffer used in the synthesis of **4** was supplemented with soluble media components, individually prepared for the two bacterial strains (see experimental section for details). This avoids a deleterious dilution of growth media during fermentation, even at high concentrations of **4**. It is important to note that **4** was synthesized in its racemic form, whereas PKS exclusively accept (*S*)-MMCoA. This was shown for the biosynthesis of erythromycin and is assumed to be analogous for homologous systems [7]. Thus, the concentration of (*S*)-**4** that is available for the PKS is half of the fed concentration.

**Figure 3:**
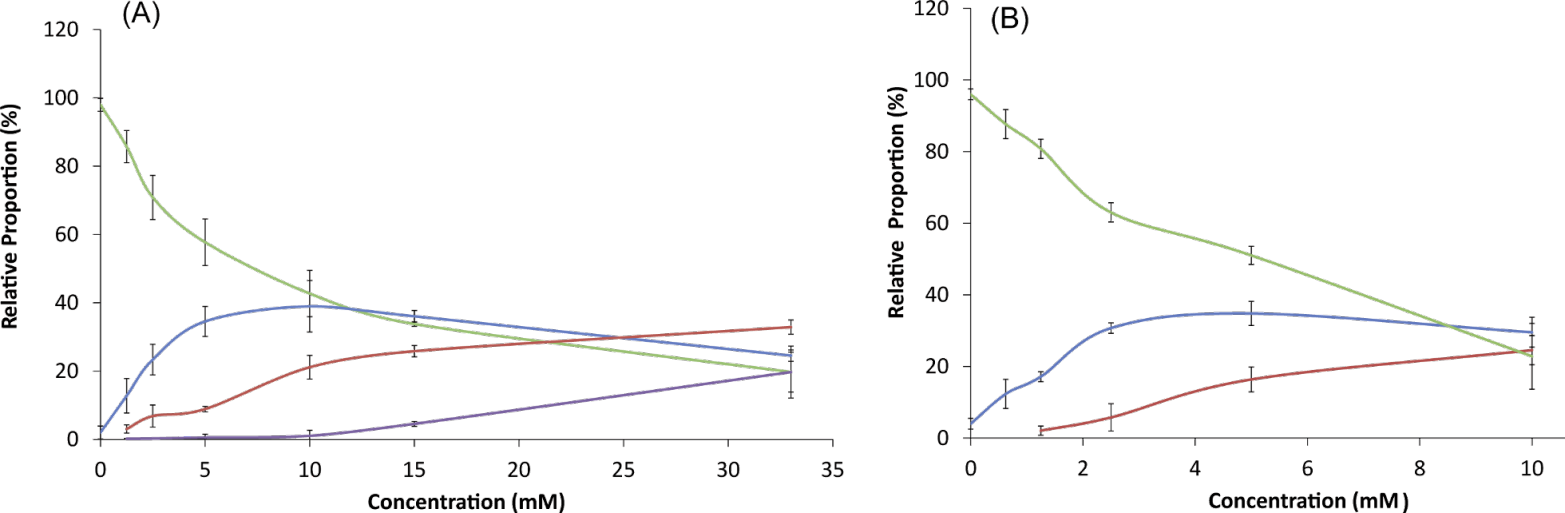
Relative incorporation of the D_3_-label into erythromycin (A) and rapamycin (B), depending on the fed concentration of *rac*-**4**. Color coding: green: no incorporation; blue: single; red: double; magenta: triple incorporation as detected by LC/ESI–MS.

To analyze the incorporation of D_3_-methylmalonyl-SNAC into the erythromycin biosynthesis, fermentations were carried out in triplicate in 24-well plates by using the Duetz system, allowing for small-scale and reproducible fermentations (see experimental section for details on the fermentation system) [42]. In contrast, fermentation for the incorporation of D_3_-methylmalonyl-SNAC into rapamycin biosynthesis was carried out in 50 mL Erlenmeyer flasks, as fermentation of this polyketide in deep-well plates was found to be poorly reproducible. Compound **4** was added in a series of concentrations to both fermentations, and the incorporation was quantified (Figure 3).

#### Erythromycin

**Figure 4:**
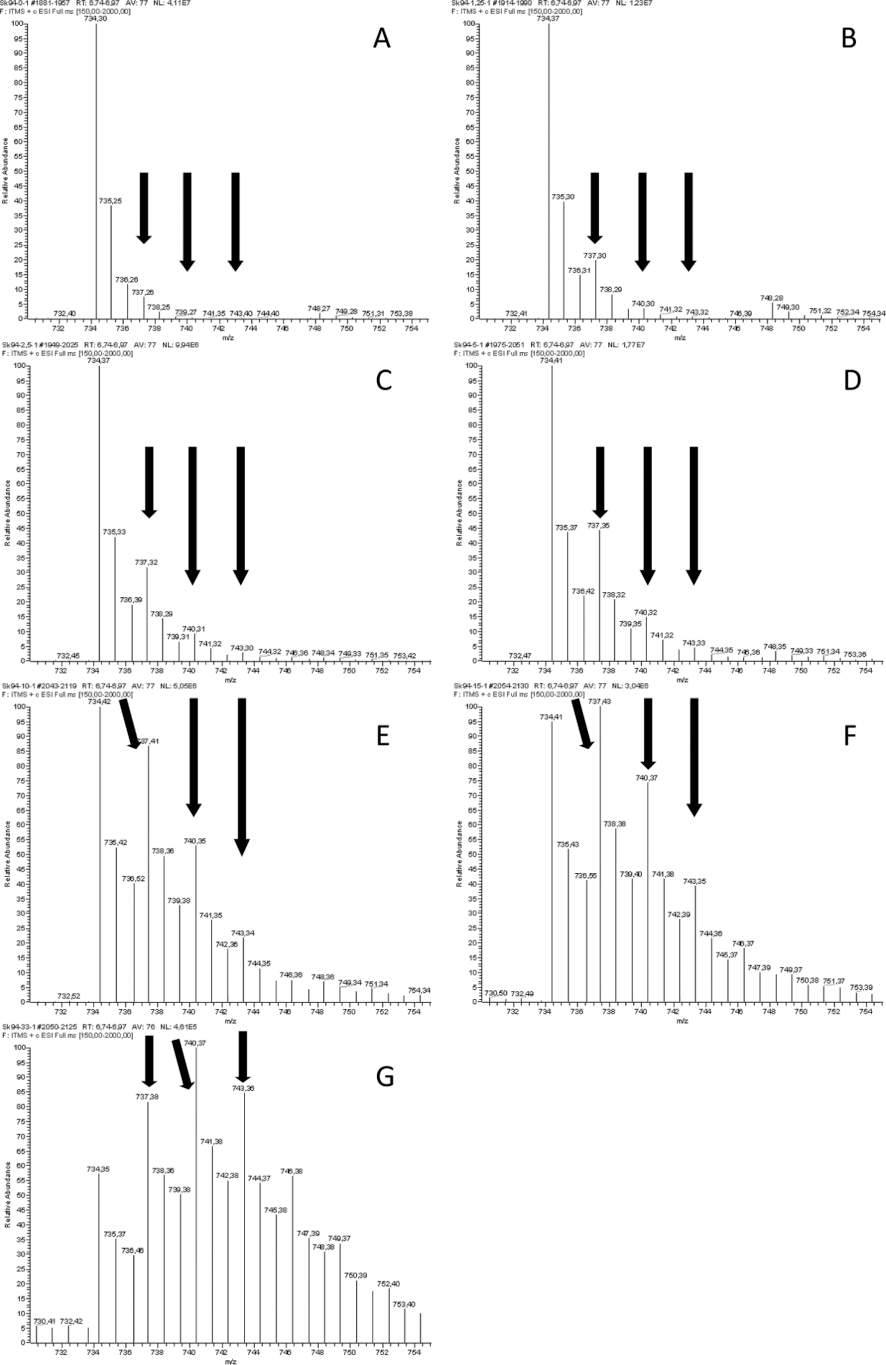
ESI–MS spectra of feeding experiments with an erythromycin-producing culture of *S. erythraea*. The mass spectra show the change in the isotope ratio of the detected erythromycin, depending on the concentration of **4** in the fermentation medium. (A) control, 0 mM **4**; (B) 1.25 mM **4**; (C) 2.5 mM **4**; (D) 5 mM **4**; (E) 10 mM **4**; (F) 15 mM **4**; (G) 30 mM **4**. The data are quantified by subtraction of the intensity of the mass for all relevant ions (beginning from 734.3 in steps of three, corresponding to the D_3_-label) of the control from the experiments. An analogous figure (Figure S9) for the feeding studies with a rapamycin-producing culture of *S. hygroscopicus* can be found in Supporting Information File 1.

It was observed that with the exogenous racemic malonate **4** supplied at a concentration of 1.25 mM, a single building block out of six endogenous methylmalonyl-CoA building blocks, was replaced in ~17% of the detected erythromycin. Additionally, at concentrations higher than 2.5 mM, a second exogenously supplied building block was incorporated, and a third at concentrations above 10 mM. At a concentration of 33 mM a triple incorporation was observed in ~20% of the detected erythromycin, measured after 5 days of fermentation (see Figure 3A and Figure 4 for MS spectra). A randomized distribution over all six MMCoA-utilizing DEBS-modules can be assumed. With this detection method, the random incorporation of the D_3_-label was measured over all six modules of the erythromycin PKS.

#### Rapamycin

In the case of rapamycin, feeding was carried out at a maximal concentration of 10 mM. At this concentration no triple incorporation was detectable. The single incorporation was observed in the same way as for the erythromycin feeding study at a concentration of 1.25 mmol of **4** in ~13% of the detected rapamycin. The second incorporation was observed at a concentration higher than 2.5 mmol. Again, the incorporation was observed over the whole five days of the fermentation and it can be assumed that the incorporation is randomly distributed over all seven MMCoA-utilizing modules (out of 14 in total) of the rapamycin PKS (RAPS). This indicates a similar efficiency of the whole incorporation process, beginning with membrane transfer and ending with the trans-thioesterification onto the enzyme. Consequently, the exogenous MM-SNAC can actually compete with MM-CoA if provided in millimolar concentrations. Through the relatively straightforward synthetic access to this compound, these concentrations are easily reachable and can be used to overcome the expectedly weaker productivity inside the PKS machinery.

In order to quantify a potential in vivo replacement of SNAC with the native activator coenzyme A, which would blur the results, we directly compared the incorporation of D_3_-methylmalonate to the incorporation of **4** into the biosynthesis of rapamycin (Supporting Information File 1, Figure S8). At 2 mM feeding concentration, on average 29% of the extracted rapamycin was D_3_-labeled in the case of **4** but only 17% in the case of the non-SNAC-activated analogue. At 4 mM feeding concentration, **4** gave 44% incorporation and the analogue 31%. This corresponds to a 1.5- to 1.7-fold more efficient incorporation upon SNAC-activation. This indicates the potential incorporation of hydrolyzed and subsequently CoA-activated **4**, but clearly shows that the SNAC activation itself indeed drives the incorporation independently from further activation in vivo.

**Scheme 2:**
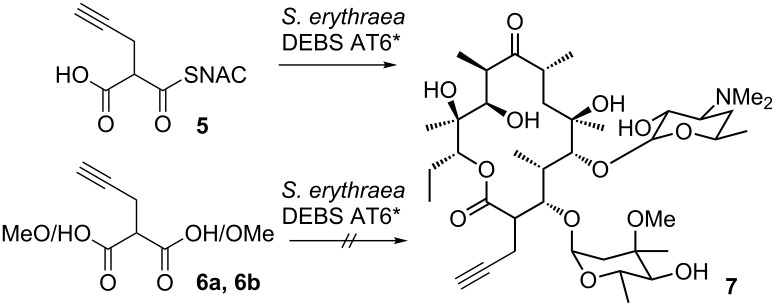
Incorporation of a propargylated malonic acid derivative into erythromycin through an active-site mutation in the acyltransferase in DEBS module 6 (DEBS AT6*) [39].

This is in accordance with an observation made with 2-propargylmalonyl-SNAC (**5**), whose acceptance by a specific acyltransferase variant in the erythromycin PKS leads to the formation of 2-propargylerythromycin A (Scheme 2) [39]. In that experiment, the active site of DEBS AT6 was redesigned to increase acceptance of **5** for incorporation into erythromycin A. A targeted mutation was introduced into the erythromycin producer *S. erythraea*. The wild-type strain showed no incorporation of **5** over the background signal. However, when the mutation Val295Ala was introduced into DEBS AT6, a significant fraction of the complete erythromycin A production was redirected towards the generation of 2-propargylerythromycin A [39].

The feeding studies for that experiment were carried out in fermentations of *S. erythraea*, thereby utilizing the full productivity of this microorganism at the expense of overcoming its complexity. When 2-propargylmalonic acid (**6a**) or its dimethyl ester **6b** were fed to the culture of *S. erythraea* DEBS AT6*, no incorporation into erythromycin was observed. Furthermore, the low catalytic promiscuity of wild type MatB malonyl-CoA synthetases was recently described [40], pointing out the need for an artificial and versatile activation mechanism as described here, to enable what we have termed enzyme-directed mutasynthesis.

## Conclusion

Based on the single incorporation of **4** a relative incorporation rate of around 40% could be observed at concentrations below 10 mM. When the substrate concentration is increased the single incorporation stagnates or decreases; instead double- and even triple-incorporation is observed in amounts increasing with the concentration of **4.** Overall up to ~60% of erythromycin and rapamycin is D_3_-labelled to a varying extent in the presence of 10 mM *rac*-**4**, corresponding to 5 mM of the naturally accepted (*S*)-enantiomer.

Significant incorporation of SNAC-activated methylmalonate **4** into the biosynthesis of erythromycin and rapamycin requires no significantly different concentrations of (*S*)-**4** between the two fermentations (typically ≥1 mM). This indicates a sufficient reactivity with PKS. However, when considering that the compound competes with endogenous MM-CoA, which is present at micromolar concentration in actinomycete cells [43], it is apparent that analogous malonate derivatives will have to be supplied in large amounts to ideally enable saturation of acyltransferase variants with artificial building blocks. It also has to be taken into account that **4** has to migrate from the growth medium into the intracellular environment before reacting with the PKS; currently no statement on the intracellular concentration of the artificial building block can be made.

The incorporation of non-native biosynthetic building blocks into polyketide biosynthesis is an emerging field of research and requires either the elaborated engineered biosynthesis of such a building block in the host or the straightforward supply with an exogenous SNAC-activated malonate. However, this feeding has not yet been systematized, and especially the competition of the non-native thioester activation with the endogenous coenzyme A had yet to be quantified. We here show that *N*-acetylcysteamine is indeed competitive to coenzyme A in its ability to activate methylmalonate for utilization by polyketide synthases. Interestingly, the efficiency of incorporation is comparable between two rather different polyketide producers from two different bacterial genera, indicating a potentially more general trend in the values measured in this study.

Knowledge about the concentrations of SNAC-activated building blocks required for feeding experiments under conditions optimal for polyketide fermentation is critical, as unnecessary consumption of the rather expensive material should be avoided, as should an underdosage, leading to false-negative outcomes or low yields. The results obtained clearly show the benefit of providing an activated building block, which is especially relevant for non-native substances, but apparently also in the case of pseudo-native building blocks as the cellular machinery is not sufficiently efficient to achieve maximal incorporation rates.

Overall, this study serves as a knowledge source for future experiments aiming at the incorporation of non-native building blocks into PKS. The efficiency of SNAC as a CoA-mimic is demonstrated in a direct competition at low millimolar concentrations of (*S*)-**4** and seems to be transferable between different species and enzymes, hence providing a good starting point for individual optimizations.

## Experimental

### General

Unless otherwise stated, materials for chemical synthesis were obtained from commercial suppliers (Sigma-Aldrich, Alfa Aesar, Fluka, Acros) in the highest purity available and used without further purification. Dry solvents were purchased from Sigma-Aldrich, stored over molecular sieves and used as supplied. Solvents used for extraction and chromatography were purchased from Thermo Fisher Scientific. Flash chromatography was carried out using Acros silica gel 60 (35–70 µm mesh). Thin-layer chromatography (TLC) was performed on aluminium-backed, precoated silica gel (60 F_245_) from Merck with cyclohexane/EtOAc or DCM/MeOH mixtures as mobile phases. Spots were detected by staining with KMnO_4_ solution (5.0 g KMnO_4_, 33 g K_2_CO_3_, 10 mL 5% aqueous NaOH in 500 mL H_2_O) and subsequent heat treatment.

NMR spectra were recorded by using a Varian Mercury 400 (400 MHz, ^1^H; 100 MHz, ^13^C) spectrometer and calibrated using residual undeuterated solvent as an internal reference. High-resolution mass spectra were recorded at LTQ Orbitrap with Accela HPLC-System (column Hypersil Gold, length 50 mm, inside diameter 1 mm, particle size 1.9 µm, ionization method: Electrospray Ionization). Products were characterized by NMR (^1^H, ^13^C) and HRMS.

### ESI-method for quantification of D_3_-label incorporation

Mass-to-charge ratios of the extracts were analyzed by HPLC coupled to a mass spectrometer. The separations were carried out on an Accela HPLC-System (consisting of pump, autosampler, column oven and PDA detector) coupled online to an Orbitrap mass spectrometer equipped with a LTQ XL linear ion trap (Thermo Electron Corporation, Dreieich, Germany) using the standard electro spray ionization source. Parallel UV absorption was detected at 210 and 254 nm. All solvents were LC–MS grade (Chromasolv, Sigma-Aldrich, Munich, Germany). A 5 µL amount of each sample was injected by an autosampler (*T* = 10 °C) onto a CC12514 Nucleor C18 Gravity column (3 µm particle size; Macherey-Nagel Germany) using a flow rate of 500 µL/min.

Erythromycin: A linear gradient starting with 80% solvent A/20% solvent B for one minute and increasing to 0% solvent A/100% solvent B in 10 min. After that the column was washed with 0% solvent A/100% solvent B for 5 min and re-equilibrated to the starting conditions for an additional 5 min (solvent A: water containing 0.1% formic acid; solvent B: acetonitrile containing 0.1% formic acid).

Rapamycin: A linear gradient starting with 18% solvent A2/82% solvent B2 for one minute and increasing to 0% solvent A2/100% solvent B2 in 10 min. After that the column was washed with 0% solvent A2/100% solvent B for 5 min and re-equilibrated to the starting conditions for an additional 5 min (solvent A2 water containing 10 mM NH_4_OH, solvent B2 methanol containing 10 mM NH_4_OH and 125 mL THF/2.5 L).

For mass spectrometric detection the electrospray ionization was carried out in positive (erythromycin)/negative (rapamycin) ionization mode by using a source voltage of 4 kV. The capillary voltage was set to 18 V, the capillary temperature to 275 °C, and the tube lens voltage to 115 V. Spectra were acquired in full scan centroid mode with a mass-to-charge range from 200 to 2000.

### Synthesis

**Synthesis of lithium diisopropylamine (LDA):** Diisopropylamine (2 mL,14.23 mmol) (freshly distilled from NaH) was mixed with 5 mL abs. THF under Ar. The solution was cooled to −78 °C. Then 9.78 mL (15.65 mmol) *n*-butyllithium (1.5 M solution in hexane) was added dropwise, and the reaction mixture was stirred for 40 min at −78 °C. After 40 min the white suspension was allowed to reach room temperature and the clarified solution was used immediately.

**Synthesis of *N*-acetylcysteamine (SNAC)** [25]: Cysteamine hydrochloride (11.4 g, 100 mmol), 25.2 g (300 mmol) NaHCO_3_ and 5.6 g (100 mmol) KOH were added to 500 mL of deionized H_2_O. After everything was dissolved, 9.5 mL (100 mmol) acetic anhydride was added dropwise. After stirring at room temperature for 2 h, the light rose solution was brought to pH = 4 with conc. HCl and extracted three times with 100 mL EtOAc. The combined organic layers were dried over Na_2_SO_4_ and purified by column chromatography (DCM/MeOH 99:1) to obtain 7.56 g (61%) of the desired product as a colorless oil. *R*_f_ 0.42 (DCM/MeOH 9:1); HRMS (GC–MS): [M + H]^+^ calcd for C_4_H_10_ONS, 120.04776; found, 120.04730; ^1^H NMR (400 MHz, CDCl_3_-*d*_1_) 1.34–1.38 (t, *J* = 8.4 Hz, 1H), 1.97 (s, 3H), 2.60–2.66 (m, 2H), 3.36–3.40 (m, 2H), 6.33 (bs, 1H); ^13^C NMR (101 MHz, CDCl_3_-*d*_1_) 23.1, 24.5, 42.6, 170.5.

**Synthesis of 3-*tert*-butoxy-2-D_3_-methyl-3-oxopropanoic acid (2):** 3-*tert*-Butoxy-3-oxopropanoic acid (500 mg, 3.12 mmol) (freshly purified by column chromatography before use) was dissolved in 10 mL abs. THF under Ar. The solution was cooled to −78 °C. Subsequently, 7.17 mmol of freshly prepared LDA were slowly added and the resulting mixture was stirred for 15 min. Then the cooling bath was removed and 433 mg (190 µL, 3.12 mmol) D_3_-MeI was added dropwise. Afterwards the brown reaction mixture was stirred for 18 h at rt, then cooled to 0 °C and quenched with 5 mL saturated NH_4_Cl solution. The organic layer was recovered and the solvent removed in vacuo. The resulting slurry was diluted with 50 mL saturated NaHCO_3_ solution and washed with 50 mL of EtOAc. The organic layer was washed twice with 50 mL sat. NaHCO_3_ solution. The combined water phase was acidified to pH = 1 with conc. HCl and extracted three times with 50 mL EtOAc. The combined organic layers were dried over Na_2_SO_4_, and the crude product was purified by column chromatography (cyclohexane/EtOAc 95:5) to obtain 200 mg (37%) of **2** as a slightly yellow oil. *R*_f_ 0.5 (DCM/MeOH 9:1); HRMS (GC–MS): [M + H]^+^ calcd for C_8_H_12_^2^H_3_O_4_, 178.11532; found, 178.11512; ^1^H NMR (400 MHz, CDCl_3_-*d*_1_) 1.46 (s, 9H), 3.36 (s, 1H); ^13^C NMR (101 MHz, CDCl_3_-*d*_1_) 21.1, 28.2, 47.0 ,82.7, 169.6, 176.6.

**Synthesis of *tert*-butyl 3-((2-acetamidoethyl)thio)-2-D_3_-methyl-3-oxopropanoate (3):** 810 mg (4.65 mmol) of compound **2** were dissolved in 10 mL abs. THF under Ar. Subsequently, 981 mg (6.05 mmol) CDI and 170 mg (1.40 mmol) DMAP were added at 0 °C, and the mixture was stirred for 45 min at 0 °C before 1.109 g (9.30 mmol) SNAC was added dropwise. The reaction mixture was stirred for another 30 min at 0 °C and then for 18 h at rt. The solvent was removed in vacuo and the residue was suspended in 50 mL 0.1 M HCl. The suspension was extracted three times with 50 mL of DCM. The combined organic layers were collected, dried over Na_2_SO_4_, and purified by column chromatography (DCM/MeOH 99:1) to obtain 1.05 g (82%) of **3** as a slightly yellow oil. *R*_f_ 0.5 (DCM/MeOH 9:1); HRMS (LCMS–ESI): [M + H]^+^ calcd for C_12_H_19_^2^H_3_O_4_NS, 279.14524; found, 279.14567; [M + Na]^+^ calcd for C_12_H_19_^2^H_3_O_4_NSNa, 301.12718; found, 301.12749; ^1^H NMR (400 MHz, CDCl_3_-*d*_1_) 1.39 (s, 9H), 1.90 (s, 3H), 2.98–3.02 (m, 2H), 3.32–3.39 (m, 2H), 3.48 (s, 1H), 6.34 (bs, 1H); ^13^C NMR (101 MHz, CDCl_3_-*d*_1_) 23.2, 27.8, 28.8, 39.6, 54.9, 82.4, 168.6, 170.5, 196.8.

**Synthesis of 3-((2-acetamidoethyl)thio)-2-D_3_-methyl-3-oxopropanoic acid (4):** 513 mg (1.84 mmol) of compound **3** were dissolved in 50 mL abs. DCM under Ar. At 0 °C 873 mg (505 µL, 4.66 mmol) TiCl_4_ was added dropwise. The dark brown reaction mixture was stirred for 30 min at 0 °C, then for another 6 h at room temperature. After 6 h (DC-control) the reaction mixture was quenched with 19 mL of the individual NaHCO_3_-based feeding buffer (see below) in an ice bath to reach a final concentration of 0.1 M of the product. DCM was evaporated at room temperature and the remaining white slurry was transferred to 50 mL polypropylene tubes. After centrifugation for 4 min to remove TiO_2_, the resulting clear solution was sterile filtered and used directly for feeding experiments. *R*_f_ 0.16 (MeOH/DCM 1:9); HRMS: [M + H] calcd for C_8_H_11_^2^H_3_O_4_NS, 223.08264; found, 223.08286; ^1^H NMR (400 MHz, CDCl_3_-*d*_1_) 1.95 (s, 3H), 3.10–3.13 (t, *J* = 6.2 Hz, 2H), 3.38–3.41 (t, *J* = 6.2 Hz, 2H), 3.88 (s, 1H).

### Preparation of the Feeding buffers

Erythromycin: 5 g glucose, 50 g Glucidex IT29 (Roquette), 83.83 g NaHCO_3_, and 186 mg Na_2_CO_3_ were dissolved in 1 L of Millipore water to obtain 1 M carbonate feeding buffer at pH = 8.

Rapamycin: 30 g starch (corn), 1 g yeast, 30 g Toasted Nutrisoy, 19 g Dextrin, 10 g NaCl, 83.83 g NaHCO_3_, and 186 mg Na_2_CO_3_ were dissolved in 1 L of Millipore water to obtain the 1 M carbonate feeding buffer at pH = 8.

### Feeding experiments

All experiments were performed in triplicate. Concentrations of **4** tested for incorporation in the biosynthesis of erythromycin and rapamycin were: 0 mM; 0.625 mM; 1.25 mM; 2.5 mM; 5.0 mM; 10.0 mM; 15.0 mM; and 33.33 mM; the latter two only for erythromycin. A comparison of the incorporation of **4** and D_3_-methylmalonic acid into the biosynthesis of rapamycin was carried out at 2.0 mM and 4.0 mM.

Erythromycin: *Saccharopolyspora erythraea* NRRL-B-24071 was grown on ABB13 agar [44] at 30 °C. Fermentation was performed by using the 24 deep-well format Duetz system, containing three glass beads per well. The Duetz system employs a multilayer cover for deep-well plates to ensure reproducible oxygen distribution over all wells in a plate [42]. We found the 24-well Duetz system particularly suitable for actinomycetes in contrast to standard plate covers with air-permeable foils. A 3 mL volume of TSB was inoculated with a 0.5 cm × 0.5 cm agar slice of a sporulating petri-dish culture and cultivated for 48 h at 30 °C. The main culture consisting of 3 mL SM3 [45] complemented with feeding-buffer (containing different concentrations of substrate), was inoculated with 0.125 mL preculture and incubated for 5 days at 30 °C and 180 rpm.

The cultures were transferred into a 15 mL tube and extracted with two volumes of EtOAc overnight at 18 °C. The layers were separated by centrifugation (4000 rpm, 5 min) and the supernatant was dried in vacuo. The residue was redissolved in 1 mL methanol, filtered and used for analysis.

Rapamycin: *Streptomyces hygroscopicus* NRRL 5491 was grown on SY agar [46] at 28 °C. Fermentation was carried out in 50 mL Erlenmeyer flasks containing a steel spring. A 0.5 cm × 0.5 cm agar piece was used to inoculate the preculture, 7 mL RapV7 Seed Medium [47] supplemented with 0.16 mL 20% glucose, and cultivated for 48 h at 180 rpm and 28 °C. The main culture in 7 mL MD6 medium [47] (supplemented with 0.35 mL 40% fructose and 0.1 mL L-lysine (140 mg/mL)) was mixed with feeding buffer (containing different concentrations of substrate) and inoculated with 0.35 mL preculture before incubation at 26 °C and 160 rpm for six days.

Afterwards, 10 mL methanol and glass beads were added to the culture, and the slurry was shaken for a further 3 h. The resulting suspension was transferred into polypropylene tubes and centrifuged, and 1 mL of the supernatant was filtered and used for analysis.

## Supporting Information

File 1 NMR spectra of synthesized compounds and ESI–MS spectra of D_3_-propionate incorporation.

## References

[1] Hopwood D A, Sherman D H (1990). Annu Rev Genet.

[2] Donadio S, Katz L (1992). Gene.

[3] Staunton J, Weissman K J (2001). Nat Prod Rep.

[4] Hertweck C (2009). Angew Chem, Int Ed.

[5] Khosla C, Gokhale R S, Jacobsen J R, Cane D E (1999). Annu Rev Biochem.

[6] Cortes J, Haydock S F, Roberts G A, Bevitt D J, Leadlay P F (1990). Nature.

[7] Marsden A F, Caffrey P, Aparicio J F, Loughran M S, Staunton J, Leadlay P F (1994). Science.

[8] Donadio S, Staver M J, McAlpine J B, Swanson S J, Katz L (1991). Science.

[9] Cortes J, Wiesmann K E, Roberts G A, Brown M J, Staunton J, Leadlay P F (1995). Science.

[10] McDaniel R, Kao C M, Hwang S J, Khosla C (1997). Chem Biol.

[11] Böhm I, Holzbaur I E, Hanefeld U, Cortési J, Staunton J, Leadlay P F (1998). Chem Biol.

[12] Kao C M, McPherson M, McDaniel R N, Fu H, Cane D E, Khosla C (1997). J Am Chem Soc.

[13] Kao C M, McPherson M, McDaniel R N, Fu H, Cane D E, Khosla C (1998). J Am Chem Soc.

[14] Kushnir S, Sundermann U, Yahiaoui S, Brockmeyer A, Janning P, Schulz F (2012). Angew Chem, Int Ed.

[15] Oliynyk M, Brown M J B, Cortés J, Staunton J, Leadlay P F (1996). Chem Biol.

[16] Lau J, Fu H, Cane D E, Khosla C (1999). Biochemistry.

[17] Stassi D L, Kakavas S J, Reynolds K A, Gunawardana G, Swanson S, Zeidner D, Jackson M, Liu H, Buko A, Katz L (1998). Proc Natl Acad Sci U S A.

[18] Kuhstoss S, Huber M, Turner J R, Paschal J W, Rao R N (1996). Gene.

[19] Marsden A F A, Wilkinson B, Cortés J, Dunster N J, Staunton J, Leadlay P F (1998). Science.

[20] Lowden P A S, Böhm G A, Metcalfe S, Staunton J, Leadlay P F (2004). ChemBioChem.

[21] Ridley C P, Lee H Y, Khosla C (2008). Proc Natl Acad Sci U S A.

[22] Wilson M C, Moore B S (2012). Nat Prod Rep.

[23] Mo S J, Kim D H, Lee J H, Park J W, Basnet D B, Ban Y H, Yoo Y J, Chen S-w, Park S R, Choi E A (2011). J Am Chem Soc.

[24] Quade N, Huo L, Rachid S, Heinz D W, Müller R (2012). Nat Chem Biol.

[25] Koryakina I, Williams G J (2011). ChemBioChem.

[26] Hughes A J, Keatinge-Clay A (2011). Chem Biol.

[27] Kato Y, Bai L, Xue Q, Revill W P, Yu T-W, Floss H G (2002). J Am Chem Soc.

[28] Wu J, Zaleski T J, Valenzano C, Khosla C, Cane D E (2005). J Am Chem Soc.

[29] Arnstadt K-I (1976). Justus Liebigs Ann Chem.

[30] Arnstadt K-I, Lynen F, Schindlbeck G (1975). Eur J Biochem.

[31] Kang Y K, Han S J (1997). J Phys Chem B.

[32] Miller W W, Richards J H (1968). Biochem Biophys Res Commun.

[33] Stubbe J, Fish S, Abeles R H (1980). J Biol Chem.

[34] Carroll B J, Moss S J, Bai L, Kato Y, Toelzer S, Yu T-W, Floss H G (2002). J Am Chem Soc.

[35] Khosla C, Lau J, Pohl L N (2001). Methods for making polyketides using altered PKS. WO Pat. Appl..

[36] Murli S, MacMillan K S, Hu Z, Ashley G W, Dong S D, Kealey J T, Reeves C D, Kennedy J (2005). Appl Environ Microbiol.

[37] Pohl N L, Gokhale R S, Cane D E, Khosla C (1998). J Am Chem Soc.

[38] Richardson M T, Pohl N L, Kealey J T, Khosla C (1999). Metab Eng.

[39] Sundermann U, Bravo-Rodriguez K, Klopries S, Kushnir S, Gomez H, Sanchez-Garcia E, Schulz F (2013). ACS Chem Biol.

[40] Koryakina I, McArthur J, Randall S, Draelos M M, Musiol E M, Muddiman D C, Weber T, Williams G J (2013). ACS Chem Biol.

[41] Gregory M A, Hong H, Lill R E, Gaisser S, Petkovic H, Low L, Sheehan L S, Carletti I, Ready S J, Ward M J (2006). Org Biomol Chem.

[42] Duetz W A, Rüedi L, Hermann R, O'Connor K, Büchs J, Witholt B (2000). Appl Environ Microbiol.

[43] Mo S J, Ban Y-H, Park J W, Yoo Y J, Yoon Y J (2009). J Ind Microbiol Biotechnol.

[44] Fitzgerald N B, English R S, Lampel S J, Vanden Boom T J (1998). Appl Environ Microbiol.

[45] Ranganathan A, Timoney M, Bycroft M, Cortés J, Thomas I P, Wilkinson B, Kellenberger L, Hanefeld U, Galloway I S, Staunton J (1999). Chem Biol.

[46] Wu K, Chung L, Revill W P, Katz L, Reeves C D (2000). Gene.

[47] Zhang M, Sheridan M R (2007). 39-Desmethoxyrapamycin, compositions and methods of use thereof. U. S. Patent.

