# Supplementary material for: Quantification of N-acetylcysteamine activated methylmalonate incorporation into polyketide biosynthesis
Source: Beilstein J Org Chem. 2013 Apr 5;9:664–74. doi: 10.3762/bjoc.9.75 (PMC3628877; doi:10.3762/bjoc.9.75)
Supplement: File 1 — NMR spectra of synthesized compounds and ESI–MS spectra of D3-propionate incorporation. [file Beilstein_J_Org_Chem-09-664-s001.pdf]

Supporting Information

for

Quantification of *N*-acetylcysteamine activated methylmalonate incorporation into polyketide biosynthesis

Stephan Klopries[1], Uschi Sundermann[1,2] and Frank Schulz*[1,2]

Address: [1]Fakultät für Chemie, Technische Universität Dortmund, Otto-Hahn-Str. 6, 44221 Dortmund, Germany and [2]Abteilung für Chemische Biologie, Max-Planck-Institut für Molekulare Physiologie, Otto-Hahn-Str. 11, 44227 Dortmund, Germany

Email: Frank Schulz – frank3.schulz@tu-dortmund.de

* Corresponding author

NMR spectra of synthesized compounds and ESI–MS spectra of D_3-propionate incorporation

NMR spectra of the compounds

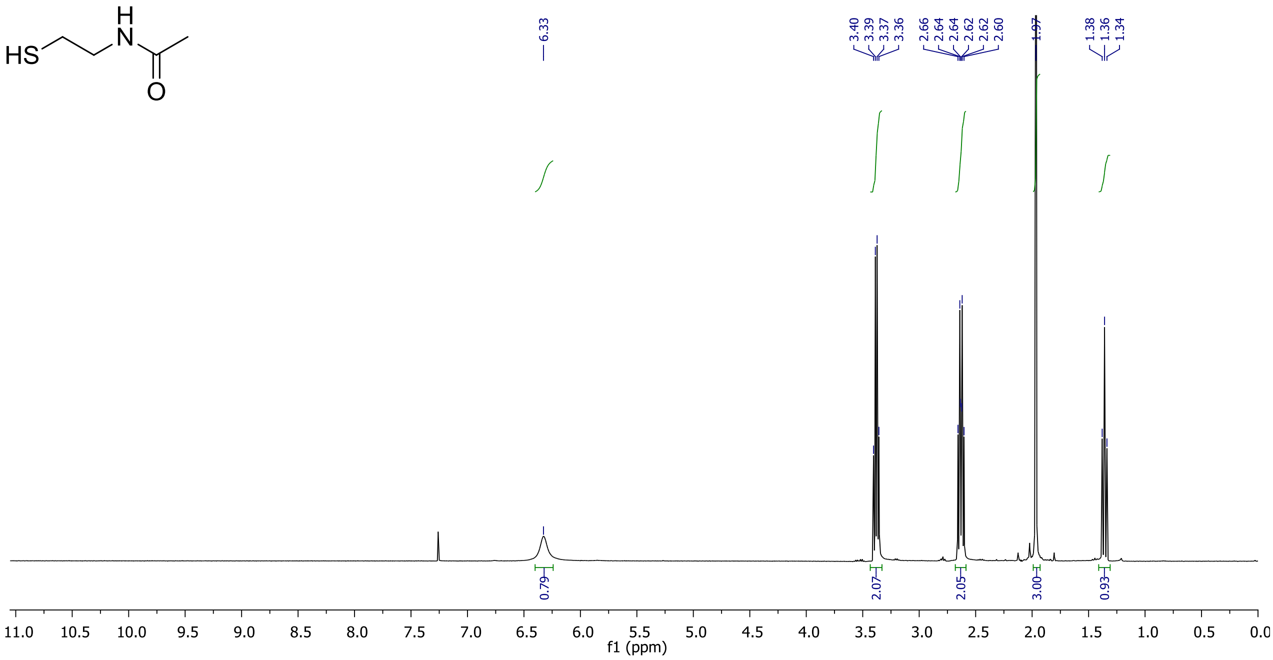

Figure S1: ^{1}H NMR spectra of SNAC in $CDCl_3$.

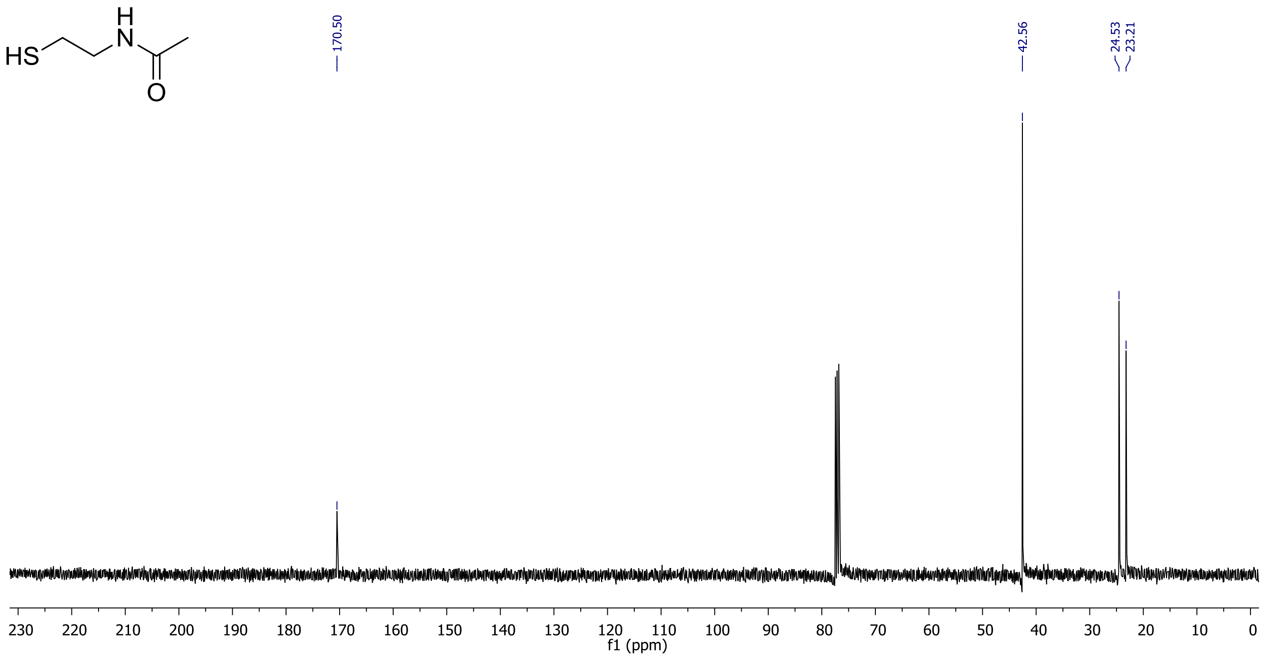

Figure S2: ^{13}C NMR spectra of SNAC in $CDCl_3$.

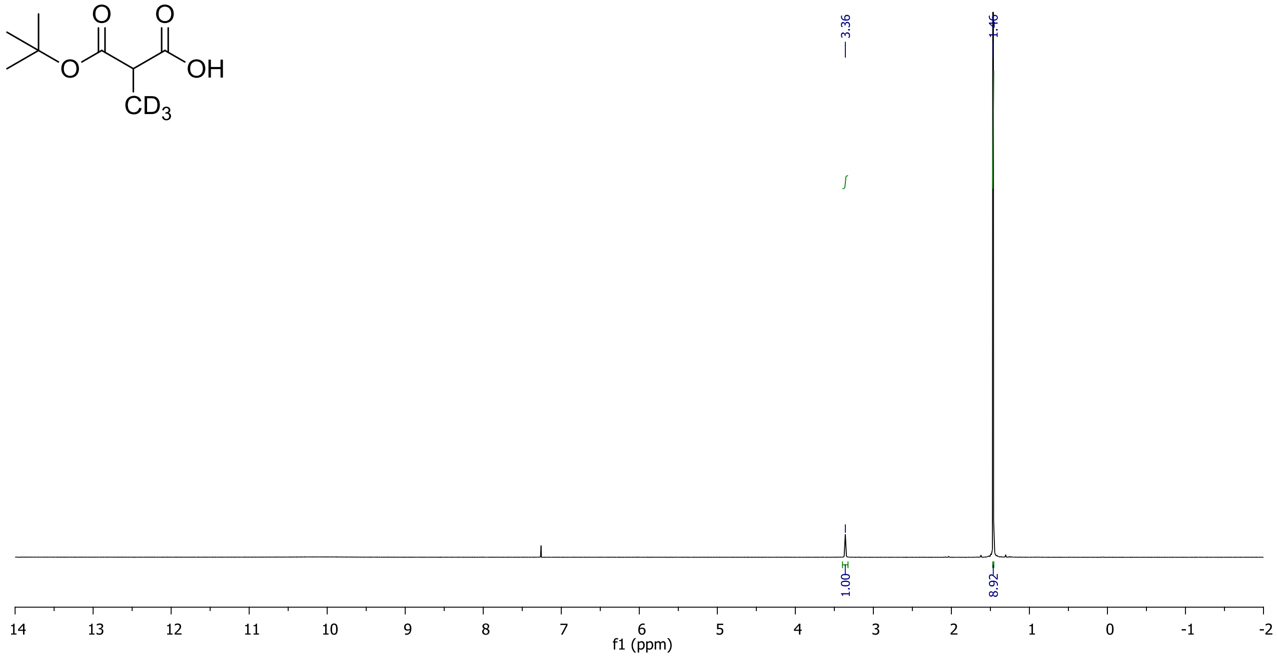

Figure S3: ^{1}H NMR spectra of 3-(*tert*-butoxy)-2-D$_3$-methyl-3-oxopropanoic acid (**2**) in $CDCl_3$.

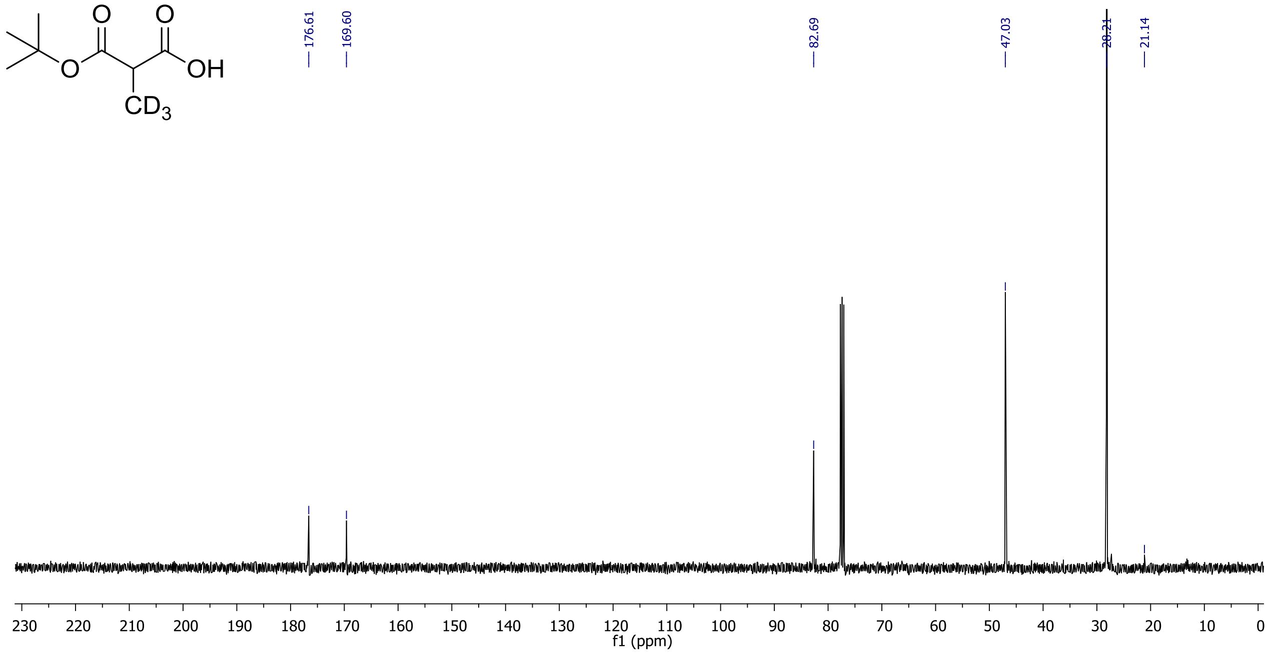

Figure S4: ^{13}C NMR spectra of 3-(*tert*-butoxy)-2-D$_3$-methyl-3-oxopropanoic acid (**2**) in $CDCl_3$.

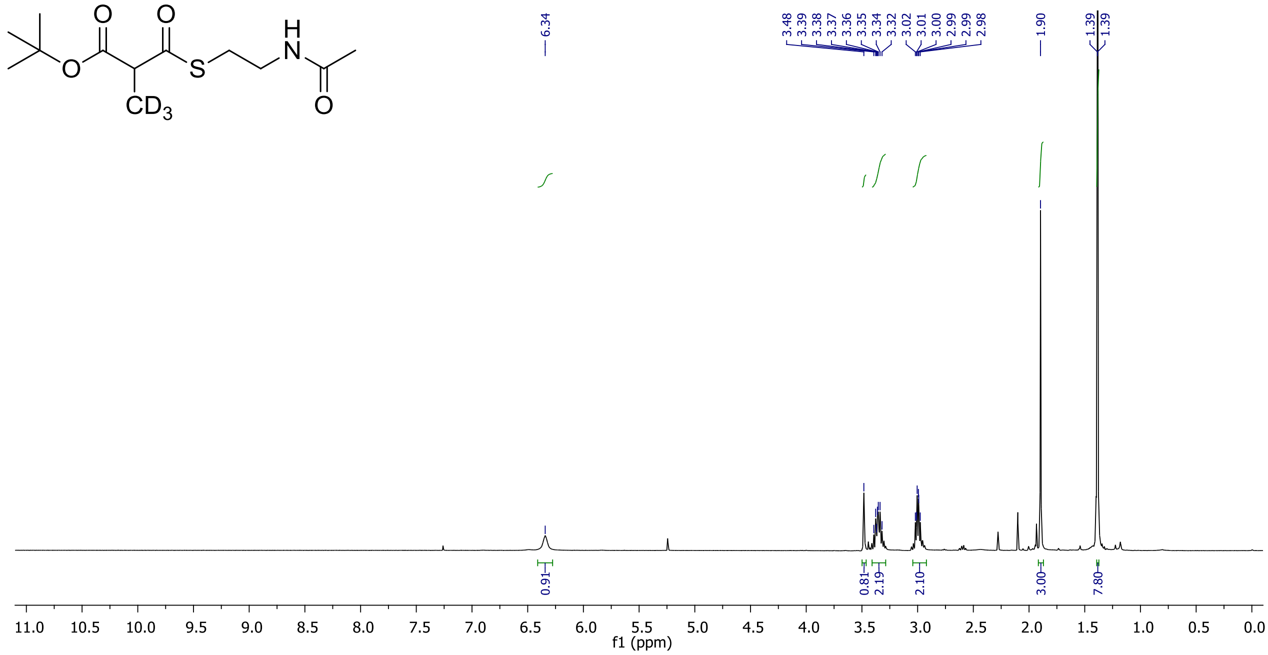

Figure S5: ^{1}H NMR spectra of *tert*-butyl 3-((2-acetamidoethyl)thio)-2-D_3-methyl-3-oxopropanoate (**3**) in $CDCl_3$.

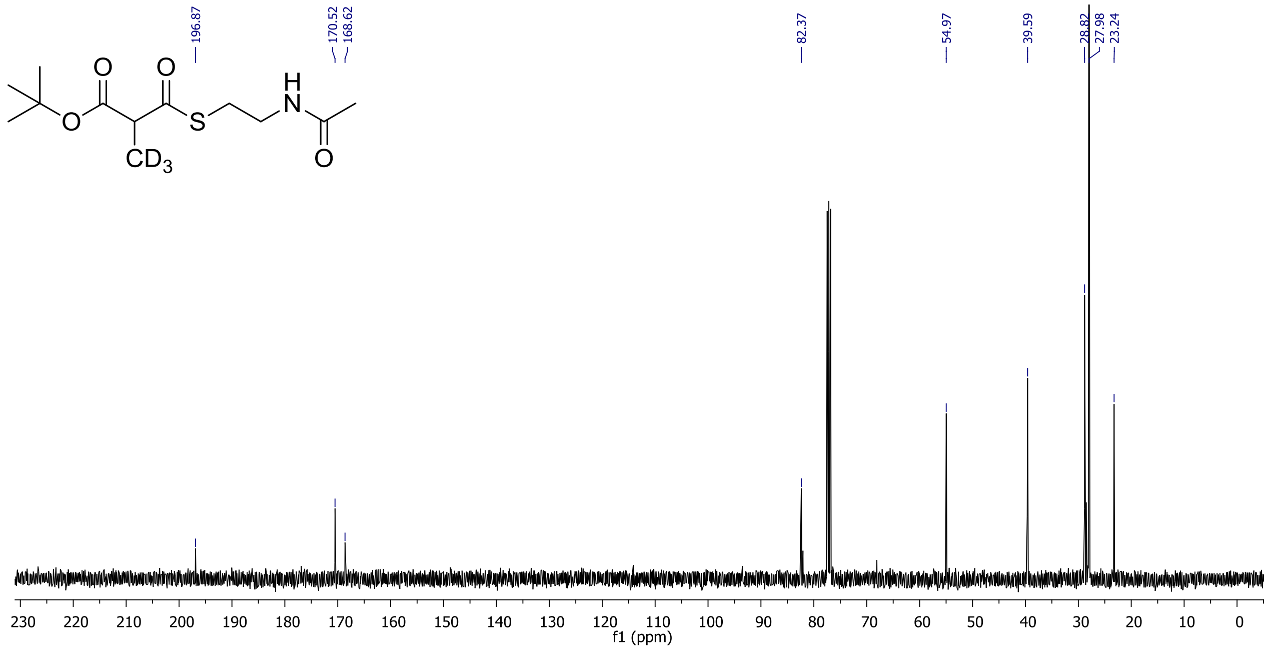

Figure S6: ^{13}C NMR spectra of *tert*-butyl 3-((2-acetamidoethyl)thio)-2-D_3-methyl-3-oxopropanoate (**3**) in $CDCl_3$.

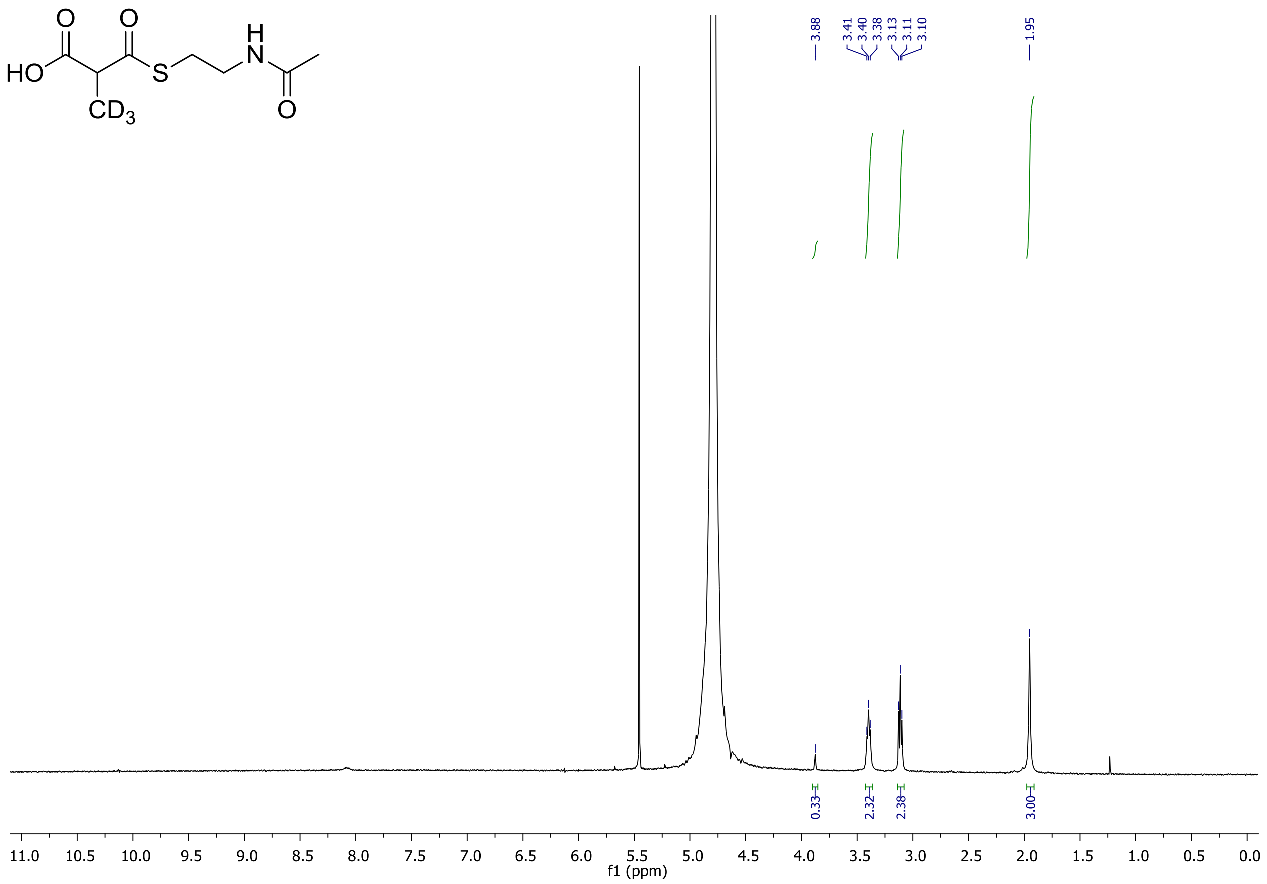

Figure S7: ^{1}H NMR spectra of 3-((2-acetamidoethyl)thio)-2-D_3-methyl-3-oxopropanoic acid (**4**) in D_2O.

Incorporation assay D_3-methylmalonic acid versus D_3-methylmalonic *N*-acetylcysteamine thioester (4)

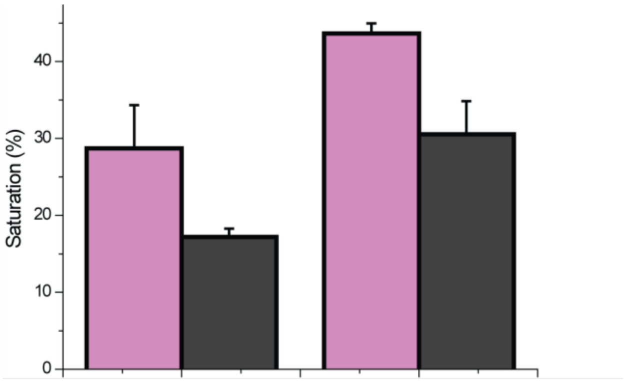

Figure S8: Relative incorporation of SNAC activated D_3-methylmalonic acid (purple) and D_3-methylmalonic acid (grey).

ESI–MS spectra of D3-labeled compounds

(A)

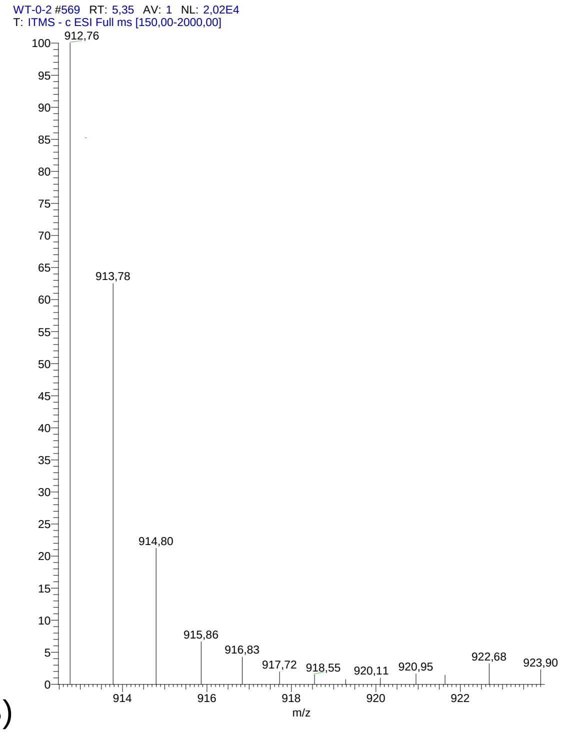

(B)

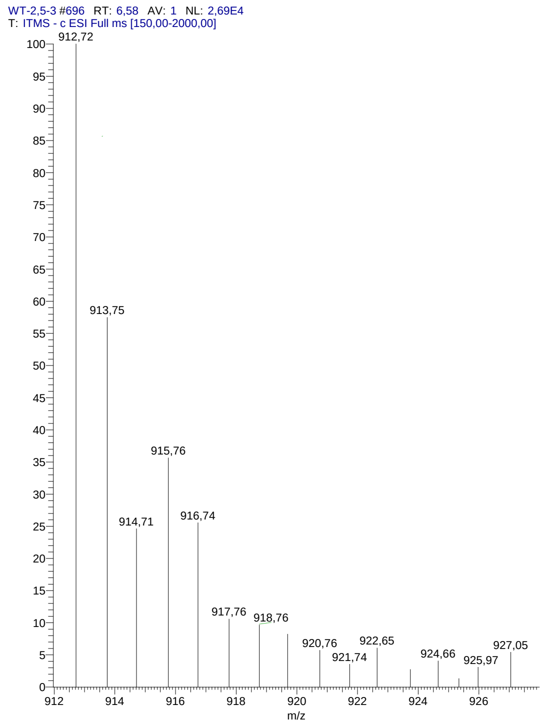

(C)

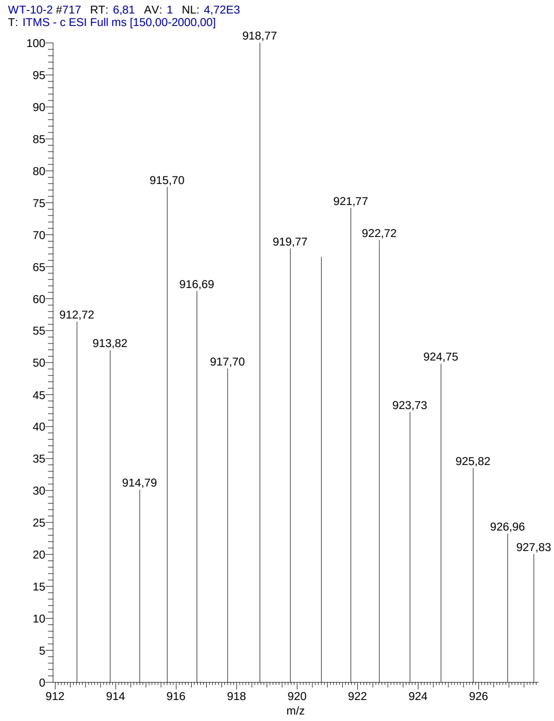

Figure S9: Showing the mass-to-charge ratio of rapamycin (HPLC/ESI–MS) after feeding the deuterated substrate at an increasing concentration. (A) 0 mM, (B) 2.5 mM and (C) 10 mM of **4**.
